# Epigenetic Alterations in Fanconi Anaemia: Role in Pathophysiology and Therapeutic Potential

**DOI:** 10.1371/journal.pone.0139740

**Published:** 2015-10-14

**Authors:** Hélio Belo, Gabriela Silva, Bruno A. Cardoso, Beatriz Porto, Jordi Minguillon, José Barbot, Jorge Coutinho, Jose A. Casado, Manuela Benedito, Hema Saturnino, Emília Costa, Juan A. Bueren, Jordi Surralles, Antonio Almeida

**Affiliations:** 1 Unidade de Investigação em Patobiologia Molecular, Instituto Português de Oncologia de Lisboa Francisco Gentil, E.P.E., Lisboa, Portugal; 2 CEDOC, Faculdade de Ciências Médicas, Universidade Nova de Lisboa, Lisboa, Portugal; 3 Laboratório de Citogenética do Instituto de Ciências Biomédicas de Abel Salazar, Porto, Portugal; 4 Center for Biomedical Network Research on Rare Diseases (CIBERER) and Department of Genetics and Microbiology, Universitat Autonoma de Barcelona, Barcelona, Spain; 5 Unidade de Hematologia Pediátrica do Centro Hospitalar do Porto, Porto, Portugal; 6 Hematopoiesis and Gene Therapy Division, CIEMAT, Madrid, Spain; 7 Serviço de hematologia do Centro Hospitalar e Universitário de Coimbra, Coimbra, Portugal; Sapporo Medical University, JAPAN

## Abstract

Fanconi anaemia (FA) is an inherited disorder characterized by chromosomal instability. The phenotype is variable, which raises the possibility that it may be affected by other factors, such as epigenetic modifications. These play an important role in oncogenesis and may be pharmacologically manipulated. Our aim was to explore whether the epigenetic profiles in FA differ from non-FA individuals and whether these could be manipulated to alter the disease phenotype. We compared expression of epigenetic genes and DNA methylation profile of tumour suppressor genes between FA and normal samples. FA samples exhibited decreased expression levels of genes involved in epigenetic regulation and hypomethylation in the promoter regions of tumour suppressor genes. Treatment of FA cells with histone deacetylase inhibitor Vorinostat increased the expression of *DNM3Tβ* and reduced the levels of *CIITA* and *HDAC9*, *PAK1*, *USP16*, all involved in different aspects of epigenetic and immune regulation. Given the ability of Vorinostat to modulate epigenetic genes in FA patients, we investigated its functional effects on the FA phenotype. This was assessed by incubating FA cells with Vorinostat and quantifying chromosomal breaks induced by DNA cross-linking agents. Treatment of FA cells with Vorinostat resulted in a significant reduction of aberrant cells (81% on average). Our results suggest that epigenetic mechanisms may play a role in oncogenesis in FA. Epigenetic agents may be helpful in improving the phenotype of FA patients, potentially reducing tumour incidence in this population.

## Introduction

Fanconi anaemia (FA) is an inherited disorder characterized by developmental abnormalities, bone marrow failure, leukemic progression and solid tumours, especially head and neck. At the cellular level, FA is characterized by impaired DNA repair and increased chromosomal fragility, a feature used in its diagnosis [1]. Mutations in 17 genes have been described, all with similar phenotypes, suggesting that all FA proteins function in common DNA repair pathway [2].

However, the severity of the disease varies even amongst patients from the same family and with the same mutation [3, 4].

It is therefore plausible that, alongside genetic mutations in FA genes, other factors may contribute to disease severity and increase the risk of neoplastic transformation.

Epigenetic modifications are important mechanisms by which cells regulate gene expression. DNA methylation and posttranslational modifications of histones affect chromatin structure, modulating gene expression and changes in cellular physiology and behavior [5]. There is ample evidence implicating epigenetic changes in the pathophysiology of MDS, AML and solid tumours [6–11]. In these malignancies, abnormal DNA methylation and histone deacetylation have been shown to silence tumour suppressor genes, and change normal expression of oncogenes, tumour suppressor genes and genes associated with several key cellular functions like DNA damage repair, cell cycle regulation, adhesion, motility, apoptosis and also signaling pathways [6, 8, 12]. For example, in ovarian and cervical cancers, hypermethylation of FANCF leads to its inactivation and to the disruption of the FA-BRCA pathway [13].

These epigenetic changes may be pharmacologically manipulated with DNA hypomethylating agents and histone deacetylase inhibitors (HDACi) [12, 14–16].

Vorinostat is an HDACi approved for the treatment of cutaneous T-cell-lymphoma. Vorinostat promotes protein acetylation, leading to the activation of genes involved in the control of cell cycle progression, differentiation and apoptosis. It also affects the expression of epigenetic regulator genes, contributing to their normal expression. In clinical trials it has shown promising clinical activity against hematological and solid tumours [17].

The aim of this study was to investigate whether epigenetic mechanisms could play a role in the pathophysiology of oncogenesis in FA and explore the potential of HDACi to improve the phenotype in FA.

## Materials and Methods

### Blood samples

Anonymized blood samples were obtained from twelve confirmed Fanconi anemia (FA) patients following written informed consent. Blood from healthy blood donors was used for normal controls. The study was approved by the Ethical Committee of Instituto Português de Oncologia de Lisboa, Francisco Gentil, EPE and all samples treated according to the Declaration of Helsinki.

### *In vitro* cell cultures

Peripheral blood mononuclear cells (PBMC) from blood samples were separated with Ficcol (Sigma) and cultured in RPMI—1640 medium supplemented with 10% fetal bovine serum (GIBCO), 2mM L-glutamine and 100μg/ml penicillin/streptomycin (all from Gibco). Treatments were performed with 1μM Vorinostat (Selleck Chemicals) or vehicle for the indicated time points.

### Gene Expression analysis by real time PCR(qPCR)

Total RNA was isolated from cells using the RNeasy Mini Kit (Qiagen), treated with DNase (Qiagen) and reverse-transcribed into cDNA using RT^2^ First Strand Kit (Qiagen) according to the manufacturer’s protocol. qPCR was performed on Roche LightCycler 480 with 84 gene specific primers for Human Epigenetic Chromatin Modification Enzymes (PAHS-085G, SABiosciences, Qiagen). Data was analyzed according to manufacturer’s instructions.

### *In silico* analysis

Bioinformatic analysis of gene expression in FA was performed using the expression array data published in Vanderwerf *I* [18].

### Analysis of DNA Methylation

Genomic DNA was extracted from primary cells (1x10^7^/ml) using Citogene kit (Citomed), treated with RNase (Citomed) and digested using EpiTect Methyl DNA Restriction Kit (Qiagen) according to the manufacturer’s protocol. qPCR was performed on Roche LightCycler 480 with 94 gene specific primers for Human Tumour Suppressor Genes (EAHS-3550ZG, Qiagen). Samples from 2 FA patients were compared with 2 Healthy donors. Data analysis was performed according to manufacturer’s instructions.

### Chromosomal instability assay

Whole blood (0,5ml) was cultured in RPMI– 1640 (supplemented as above and cultures were stimulated with 5μg/ml of phytohemaglutinin (GIBCO) during 24h. Thereafter, the cultures were treated with 1μM Vorinostat or vehicle for an additional 24h at which point 0.05 μg/ml of 1,2:3,4-diepoxybutane (DEB, Sigma) or vehicle was added to the cultures for 48h. After 96h of culture, cells were treated with 2μg/ml of colcemid (GIBCO) for 1h, spread on slides, subjected to hypotonic lysis with 75mM KCl [19, 20] and fixed in solution of 3:1 volumes of methanol:acetic acid [21].

Slides were stained with 0,5M Leishamn (Sigma) in phosphate buffer, pH 6.8. Fifty metaphases per sample were analysed for chromosome aberrations including chromosome and chromatid breaks, acentric fragments and chromosome and chromatid-type exchange. Gaps were excluded and rearrangements were scored as two breaks for the calculation of percentage of cells with aberrations.

#### Cellular viability assays

Viability was assessed by flow cytometry with Annexin-V- FITC (Biolegend) and Propidium Iodide (PI) (Sigma-Aldrich).

#### Statistical analysis

Populations were compared using unpaired 2-tailed Student’s t test or One-way ANOVA, when appropriate (a p < 0.05 was considered significant) using the GraphPad Prism version 5.00 for Windows (GraphPad Software).

## Results

The clinical characteristics of the patients whose samples were used in these experiments are detailed in Table 1.

**Table 1:** Summary of clinical data in FA patients.

Patient Number	Age (yrs)	Gender	Ethnicity	Baseline Hemoglobin	Transfusion dependence	Physical abnormalities	Solid Tumors	Current treatment
**FA.1**	**54**	**M**	**Caucasian**	**11,6**	**None**	**Short stature, café au lait spots**	**None**	**None**
**FA.2**	**18**	**F**	**Caucasian**	**13,6**	**None**	**Microcephaly, microphthalmia, short stature, Skeletal malformations**	**None**	**Escitalopram**
**FA.3**	**9**	**F**	**Caucasian**	**12**	**None**	**Short stature**	**None**	**None**
**FA.4**	**2**	**M**	**Caucasian**	**12,4**	**None**	**Congenital cardiopathy, gastrointestinal malformation, short stature, renal agenesis, hypospadias, café au lait spots**	**None**	**None**
**FA.5**	**7**	**F**	**Caucasian**	**11,4**	**None**	**Microphthalmia, short stature, café au lait spots**	**None**	**None**
**FA.6**	**36**	**F**	**Caucasian**	**11,6**	**None**	**Microcephaly, microphthalmia, short stature, café au lait spots**	**None**	**Fólic acid**
**FA.7**	**32**	**F**	**Caucasian**	**12,6**	**None**	**Microcephaly, microphthalmia, short stature, café au lait spots**	**None**	**None**
**FA.8**	**45**	**F**	**Caucasian**	**12,4**	**None**	**Microphtalmia**	**None**	**Omeprazol**
**FA.9**	**5**	**M**	**Caucasian**	**10,9**	**None**	**Microcephaly, phymosis, café au lait spots**	**None**	**None**
**FA.10**	**16**	**F**	**Caucasian**	**14,6**	**None**	**Thumb malformation, deafness, short stature, café au lait spots**	**None**	**None**
**FA.11**	**25**	**F**	**Caucasian**	**12,4**	**None**	**Microcephaly**	**None**	**None**
**FA.12**	**31**	**M**	**Caucasian**	**8**	**None**	**Slight microphtalmia**	**None**	**None**

### Fanconi anemia patients exhibit different expression of epigenetic genes compared to healthy donors

**Fig 1:**
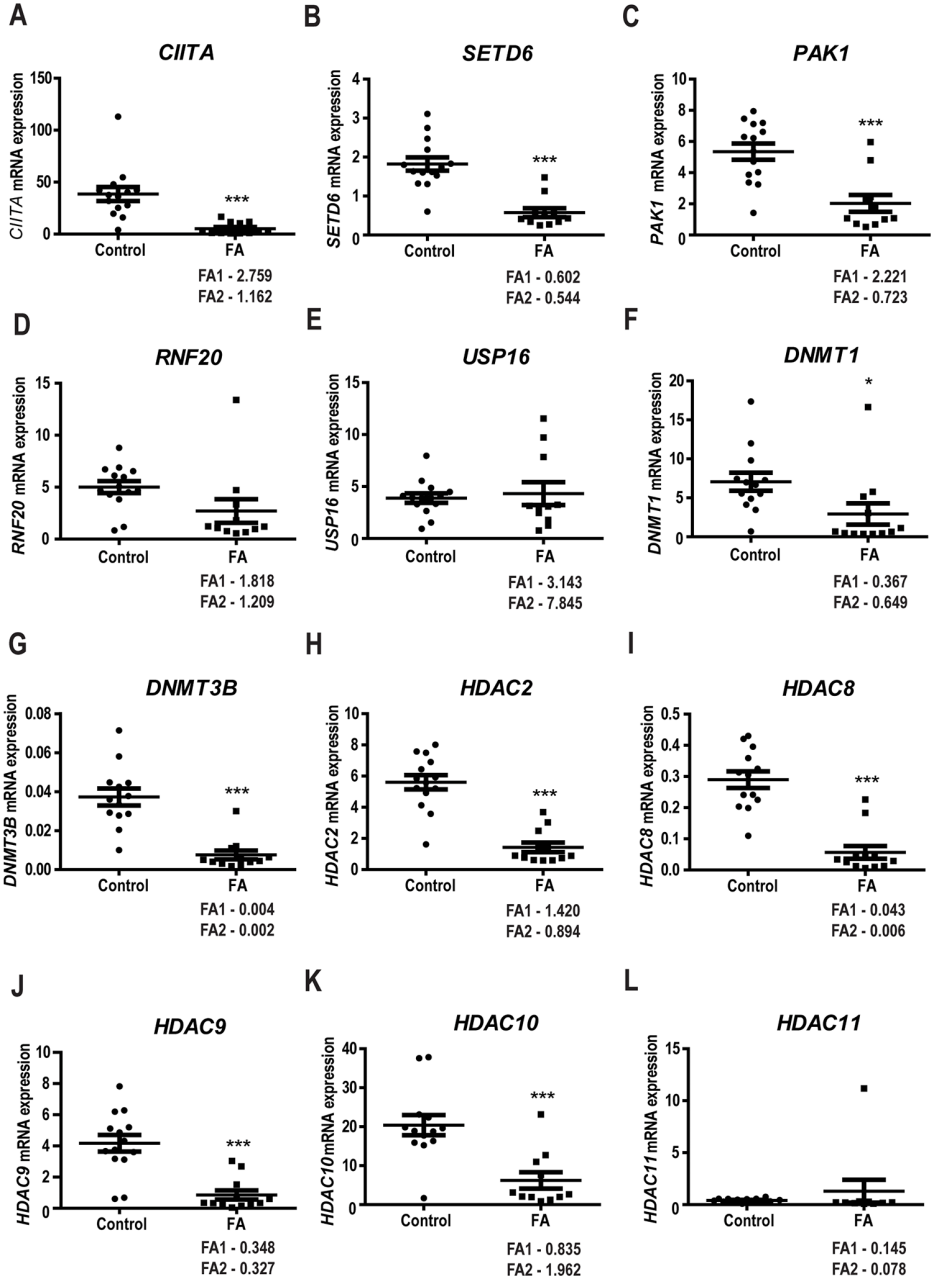
FA patients have decreased expression of epigenetic chromatin modification enzymes. RNA was isolated from PBMC from 12 FA patients and 14 healthy controls and the expression of epigenetic regulator genes was quantified using the Human Epigenetic Chromatin Enzymes PCR array (SABiosciences). Each panel (A-L) represents the expression of the indicated genes in FA patients and control samples as described in the materials and methods section.(* 0.05 >p; ** 0.01>p; *** 0.001 > p). In each panel is indicated the values of FA1 and FA2 patients whose methylation profile was also determined.

To evaluate the hypothesis that epigenetic alterations in Fanconi anemia could contribute to susceptibility to cancer, we used the Human Epigenetic Chromatin Enzymes PCR array to quantify the expression of 84 genes involved in epigenetic modification of DNA and histones in PBMC from 12 FA and compared these to PBMC from 14 healthy donors. We found that 13 genes were differentially expressed in FA as compared to normal cells (Fig 1). These included genes encoding DNA methyltransferases (DNMT1, DNM3Tβ) and genes encoding histone modifying enzymes: acetylases (CIITA), phosphorylases (PAK1), ubiquitinases (RNF20), deacetylases (HDAC2, HDCA8, HDAC9, HDAC10, HDAC11) and also methyltransferases (SETD6). This differential expression between normal and FA individuals was confirmed bioinformatically from data from Vanderwerf *et*. *al* [18] for *PAK1*, *USP16*, *DNMT1*, *DNMT3B*, *HDAC2*, *HDAC9*, *CIITA*, *HDAC10* and *HDAC11* (S1 Fig).

### FA cells exhibit DNA hypomethylation of tumour suppressor genes

**Fig 2:**
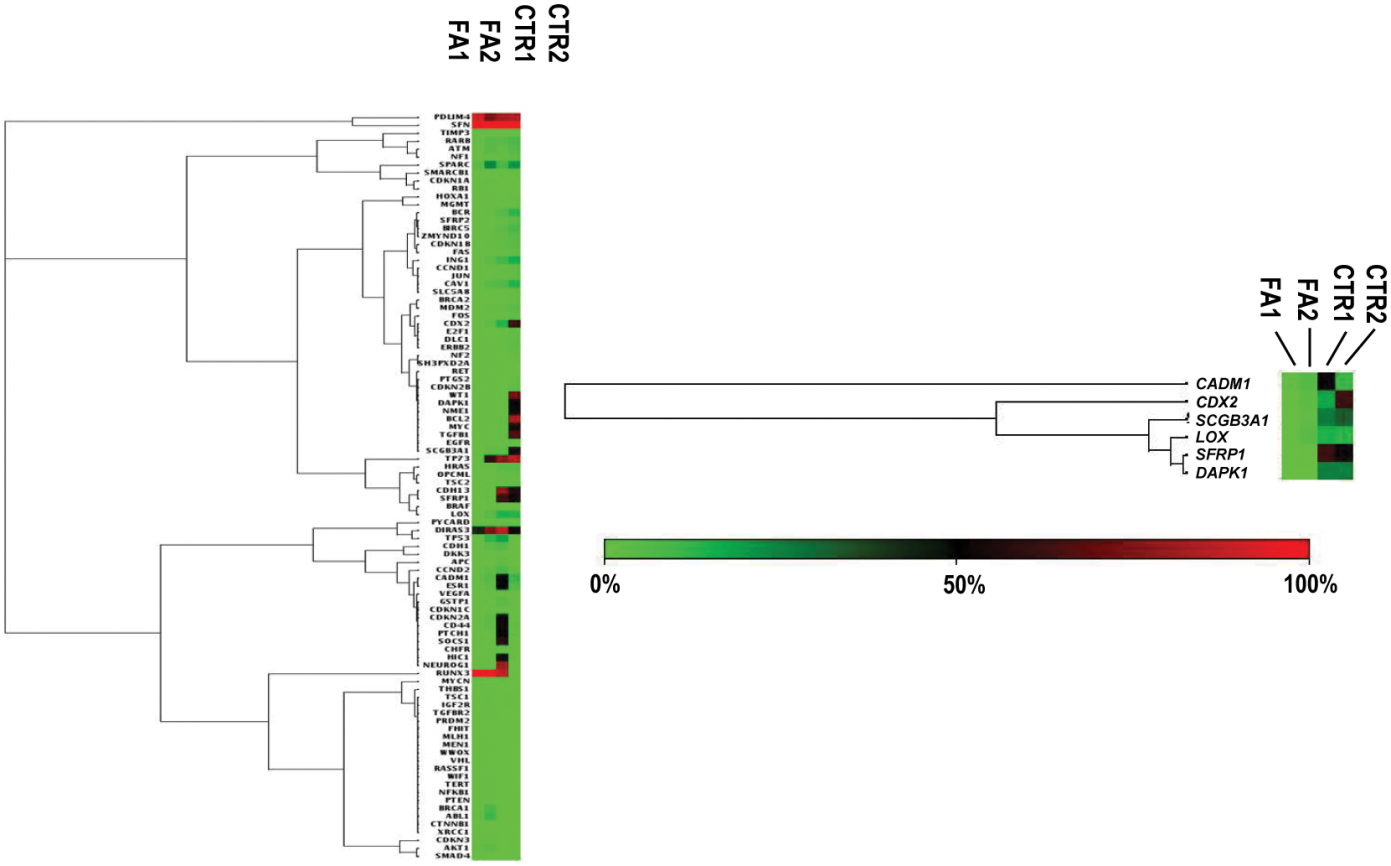
DNA methylation pattern on tumour suppressor genes in peripheral blood mononuclear cells of FA patients. PBMC from FA patients and healthy donor were isolated as in A, DNA was isolated and methylation profile was identified in 2 FA samples and 2 healthy donors samples using EpiTect Methyl II Complete PCR array (SABiosciences). Samples CTR1 and CTR2 represent control and FA1 and FA2 represent FA patients. Each row represents a tumour-supressor genes and each column represent a single DNA sample. The methylation degree are represented by the level of intensity of the square, red representing greater than 10% promoter hypermethylation, and green representing less than 10% promoter methylation (unmethylated) alleles for the tumour-suppressor gene.

In order to ascertain whether the findings from the gene expression assays translated into a difference in epigenetic patterns in FA compared to normal subjects, we studied the pattern of DNA methylation in FA PBMC. For this we used the Human Tumour Suppressor gene PCR Array to assess promoter DNA methylation of 94 tumour suppressor genes in 2 PBMC of FA patients and 2 healthy donors. This revealed a global aberrant hypomethylation of tumour suppressor genes in FA cells as compared to healthy donors. Six of 94 genes were differentially methylated in FA relative to healthy donors. These included genes whose function are related to apoptosis (*CADM1*, *SFRP1*), cell cycle (*ING1*), motility (*CDH13*), oxidative stress (*LOX*) and angiogenesis/transcription (*CDX2*) (Fig 2).

### Vorinostat modifies the expression of epigenetic genes

**Fig 3:**
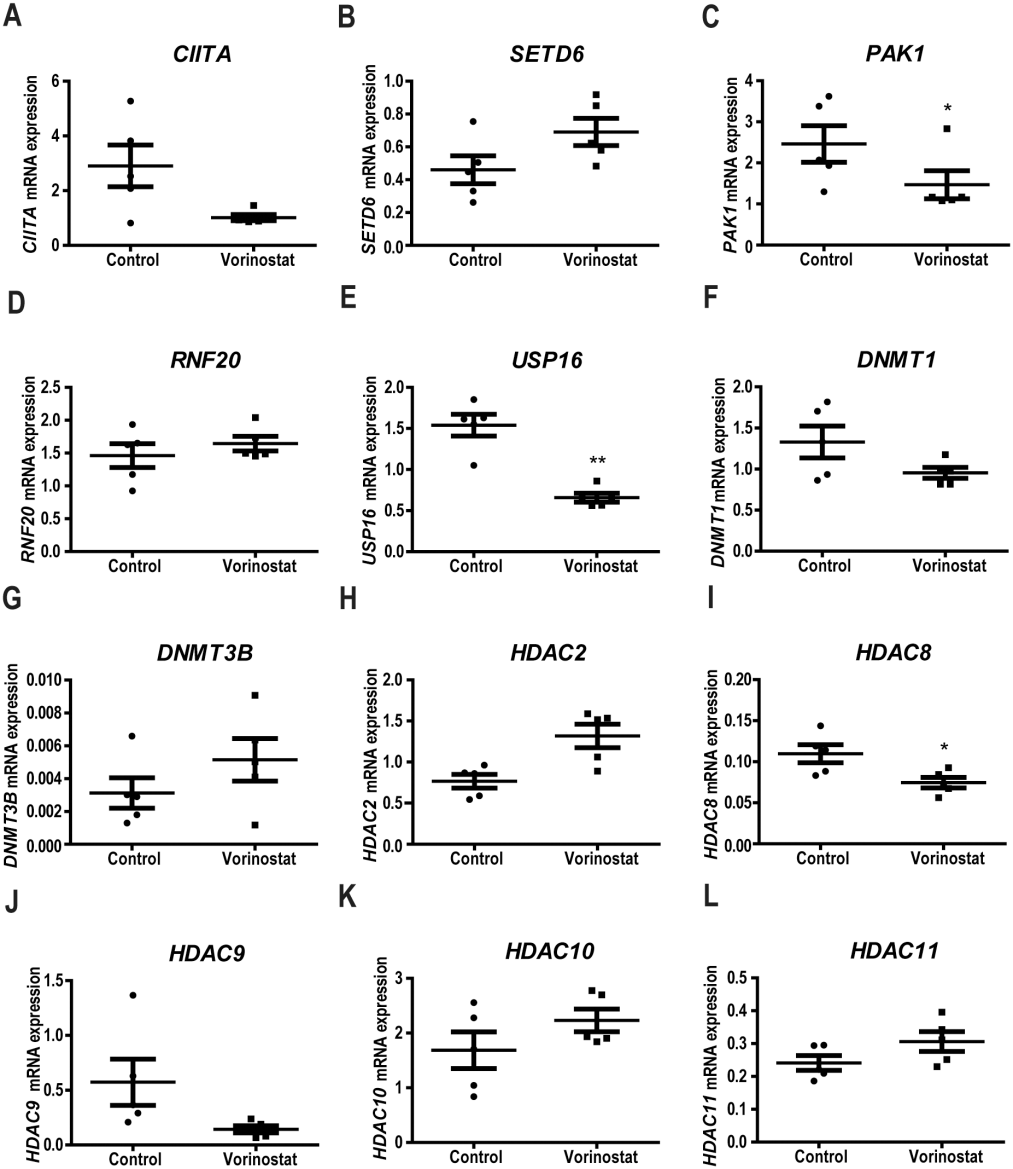
Effect of vorinostat on epigenetic patterns on chromatin epigenetic modifications enzymes differentially expressed in FA mononuclear cells. PBMC from FA patients in culture were treated with 1μM vorinostat as indicated or vehicle (control) for 8h and gene expression quantified by qPCR.

Having observed differences in epigenetic regulator gene expression and in epigenetic patterns between FA and normal subjects, we tested whether these could be normalized using epigenetic agents. We chose Vorinostat to test this effect as it is a wide HDACi which able to modulate epigenetic and gene expression patterns[8] and with proven clinical efficacy. We tested its effect on the expression of epigenetic chromatin modification genes which expression was altered in PBMC from FA patients (Fig 1). Following treatment of FA PBMC with Vorinostat for 8h and 16h (Fig 3) there was an increase in the expression levels of the *DNMT3β* gene. Interestingly, Vorinostat treatment reduced the expression of *CIITA* and *HDAC9*, involved in the immune response, *PAK1*, regulator of the *MAPK* signaling pathway, and *USP16*, involved in regulating the activity of histone H2A. The expression levels of *HDAC10*, *HDAC11*, *HDAC2*, *SETD6*, *RNF20* and *DNMT1* genes were not significantly altered by Vorinostat.

### Vorinostat reduces chromosomal breaks in FA cells

**Fig 4:**
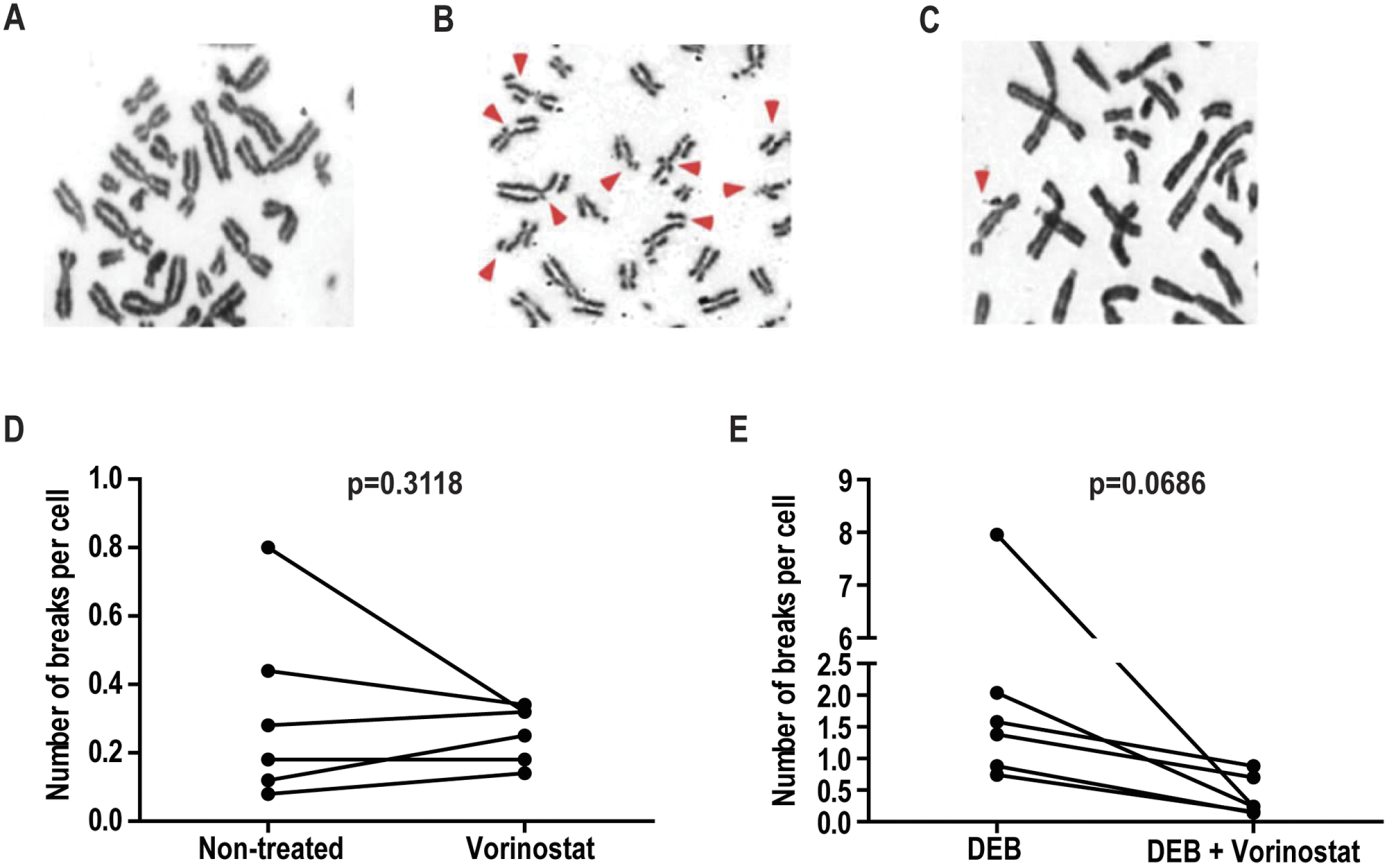
Effect of vorinostat on DEB-induced chromosome fragility of FA lymphocytes. (**A**) Lymphocytes metaphase without treatment. (**B**) Lymphocyte metaphase treated with DEB. (**C**) Lymphocyte metaphase treated with DEB after Vorinostat treatment. The red arrows indicate aberrant chromosomes characteristic of FA cells. (**D**) Number of breaks per cell after treatment with Vorinostat. (**E**) Number of breaks per cell after treatment with Vorinostat on DEB-induced breaks. The p values are indicated.

Given the capacity of Vorinostat to modulate the expression of epigenetic regulator genes in FA samples, we investigated its effect on the *in vitro* phenotype of FA cells. This functional effect of Vorinostat in FA was tested by assessing its effect on chromosome breaks induced by DEB. The percentage of aberrant cells induced by DEB was assessed on metaphases obtained from peripheral blood lymphocytes of patients with FA. Vorinostat reduced the percentage of aberrant cells (81% ± 21%, p = 0,06) in 6 patients with Fanconi anemia (Table 2, Fig 4A, 4B, 4C, 4D and 4E). There was no reduction in the number of spontaneous chromosomal breaks in FA cells following treatment with Vorinostat (Table 2).

**Table 2:** Number of breaks per cell in cultured lymphocytes from FA patients.

Mean number of breaks per cell						
Patients	Spontaneous breaks (n)	Vor 1μM	% reduction	DEB 0.05μM (n)	DEB 0.05μM + Vor 1μM (n)	% reduction
FA1	0.28 (50)	0.32 (50)	0.00	2.04 (50)	0.24 (50)	88.24
FA2	0.08 (50)	0.14 (50)	0.00	0.88 (50)	0.14 (50)	84.09
FA3	0.18 (50)	0.18 (50)	0.00	1.38 (50)	0.7 (50)	49.28
FA4	0.8 (50)	0.32 (50)	60.00	7.96 (50)	0.24 (50)	96.98
FA5	0.44 (50)	0.34 (50)	22.72	1.58 (50)	0.88 (50)	44.3
FA6	0.12 (50)	0.25 (50)	0.00	0.74 (50)	0.16 (50)	79.38
Mean±SD	0.23±0.269	0.28±0.083		1.48±2.750	0.24±0.3151	

The effect of the Vorinostat was calculated by the percentage of reduction of the number of breaks per cell in the Vorinostat treatments relatively to Spontaneous breaks and DEB.

In this experimental system Vorinostat did not significantly reduce the viability of cells (S2 Fig).

## Discussion

Fanconi anaemia is an inherited disease caused by defective DNA repair. Whereas the skeletal, genitourinary and morphological abnormalities are rarely life-threatening, the development of bone marrow failure, leukemia and solid cancers are frequently lethal. The aggressiveness of these complications are compounded by the low tolerance FA patients have to chemotherapy and radiotherapy [6]. Therefore, a treatment option which could improve the DNA repair defect and reduce the incidence of mutations and secondary malignancies would be highly desirable.

Epigenetic modifications consist in the addition or removal of small molecules onto DNA or DNA-associated proteins, i.e. histones, and enable eukaryotic cells to alter their gene expression without altering the DNA sequence [8, 22]. The molecules most commonly implicated in epigenetic regulation are methyl and acetyl groups and DNA methylation status is closely related and plays a role in regulating to histone acetylation [7, 10, 22]. In addition to the regulation of gene expression, these modifications, in particular DNA methylation, play an important role in maintaining DNA and chromatin stability [17].

Aberrant epigenetic patterns have been implicated in the pathophysiology of a variety of haematological and solid tumours [23]. These have been successfully manipulated pharmacologically with promising therapeutic results [8, 24, 25].

Our aim was to investigate whether the epigenetic machinery in FA differs from that of unaffected individuals and whether its manipulation could somehow affect the FA phenotype.

In the present study, we show that FA cells present decreased levels of several genes involved in epigenetic regulation as compared to cells from healthy subjects (Fig 1) Gene expression studies revealed that DNMT1 and DNMT3β expression, involved in DNA methylation, were significantly reduced in FA patients both in PBMCs and BM. DNMT1 is responsible for copying DNA methylation patterns established during embryonic development and their subsequent maintenance. DNMT3β is a tumour-suppressor gene with a critical role in DNA methylation playing a major role in the establishment and maintenance of genomic methylation patterns. Reduced activity of DNMT1 and DNMT3β may lead to DNA hypomethylation, inducing genomic instability and disruption of proto-oncogenes and is directly associated with tumour formation[26–28]. Abnormal expression of DNMT1 and DNMT3β has been reported in several tumours, including lung, liver, breast, ovarian, colorectal, meningiomas and lymphomas [29, 30].Reduced expression of CIITA was observed in PBMCs but not in BM. This gene regulates the expression of MHCII gene by recruiting the transcriptional machinery of basic proteins, acetyltransferases, histone deacetylases (HDAC), and other proteins involved in chromatin remodeling. CIITA expression is decreased in several types of hematological and solid tumours and is associated with decreased tumour immune recognition [31].

The expression of SETD6, a gene that may also impair anti-tumour immune response by dysregulation of the NF-kB pathway [32], was also reduced in FA PMBCs but remain roughly the same in BM.

RNF20, which is essential for the regulation of normal levels of p53 [33], is also underexpressed in FA. Its reduced expression causes chromosome instability and has been described in several types of tumours [34–48]. The decreased expression of RNF20 enhances the transcriptional effects of EGF, leading to an increase in transformation, migration and metastasis of cancer cells and tumourigenesis [5, 23, 49].

We also found reduced expression of histone deacetylases HDAC 2, 8, 9, 10 and 11 in FA PMBCs and confirmed HDAC2 and HDAC9 reduced expression in FA BM samples. HDACs regulate chromatin remodeling and regulate many genes involved both in the initiation and progression of a variety of cancers.

Despite some discrepancy between our data, obtained from blood samples and the bioinformatics analysis of gene expression data, obtained from bone marrow samples, both concur that there is altered expression of epigenetic regulating genes in FA. A larger number of samples would be required to reach firm conclusions.

The reduced expression of these genes in FA, suggests that these patients have altered epigenetic regulation, which may be involved in the neoplastic complications of this disease. This hypothesis is corroborated by our finding showing increased DNA hypomethylation at tumor suppressor gene loci in FA as compared to normal cells (Fig 2). Liu *et al* have described aberrant expression of tumor suppressor and tumor-related genes in FA, corroborating our data [50]. It is possible that this hypomethylation may increase genomic instability and have an additive effect on the DNA repair defect of FA, increasing the oncogenic potential in FA tissues.

Our initial results show that epigenetic regulation and DNA methylation are altered in FA. These findings suggested that epigenetic manipulation in FA may have a beneficial effect on this disease phenotype.

In fact, treatment of FA cells with Vorinostat induced expression of the DNMT3β, involved in the maintenance of physiological DNA methylation (Fig 3). The suppression of expression of CIITA and HDAC9 are consistent with a reduction in inflammatory response, which may play a role in oncogenesis in the context of DNA repair defects.

Of particular relevance was our finding that Vorinostat treatment reduced chromosomal breaks in FA cells (Fig 4). This may have been due to DNA stabilization through induction of DNMT3β or mediated by another mechanism yet to be investigated. Whichever mechanism at play, our findings are very suggestive that Vorinostat exerts a protective effect on the chromosomal breaks induced by cross-linking agents. This effect may have an important clinical counterpart as it may translate into greater tolerability to chemotherapeutic agents in FA patients.

These results are the first report of a potential improvement in FA phenotype with an epigenetic agent. They warrant further pre-clinical testing in animal models with a view to initiating clinical trials in FA if the results remain promising.

## Supporting Information

S1 Fig FA patients have altered expression of epigenetic chromatin modification enzymes. Each panel (**A-J**) represents the expression of selected genes of interest in FA and control samples as described in materials and methods section. With the exception of DNMT3B, there is significant difference in the expression of these genes in FA compared to normal samples (* 0.05 >p; ** 0.01>p; *** 0.001 > p).
(TIF)

S2 Fig Vorinostat has no effect on the viability of DEB-treated cells. Viability PBMC of FA patients as determined by Annexin V/PI staining following treatment with Vorinostat and DEB in cell culture medium as used to test for chromosomal fragility.
(TIF)
