# Supplementary figures and images for: Epigenetic Alterations in Fanconi Anaemia: Role in Pathophysiology and Therapeutic Potential
Source: PLoS One. 2015 Oct 14;10(10):e0139740. doi: 10.1371/journal.pone.0139740 (PMC4605638; doi:10.1371/journal.pone.0139740)

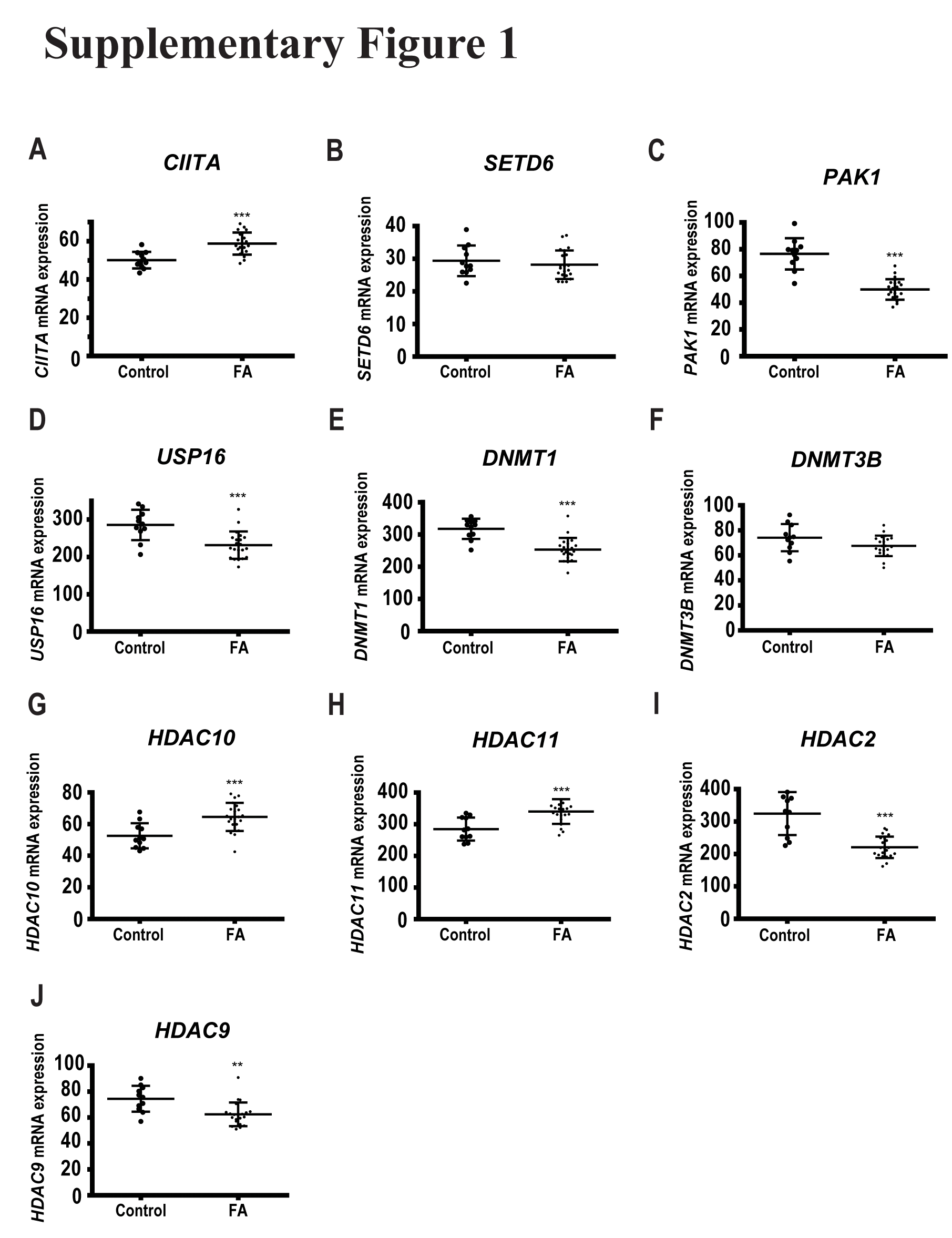

Supplement: S1 Fig — Each panel (A-J) represents the expression of selected genes of interest in FA and control samples as described in materials and methods section. With the exception of DNMT3B, there is significant difference in the expression of these genes in FA compared to normal samples (* 0.05 >p; ** 0.01>p; *** 0.001 > p). (TIF) [file pone.0139740.s001.tif]

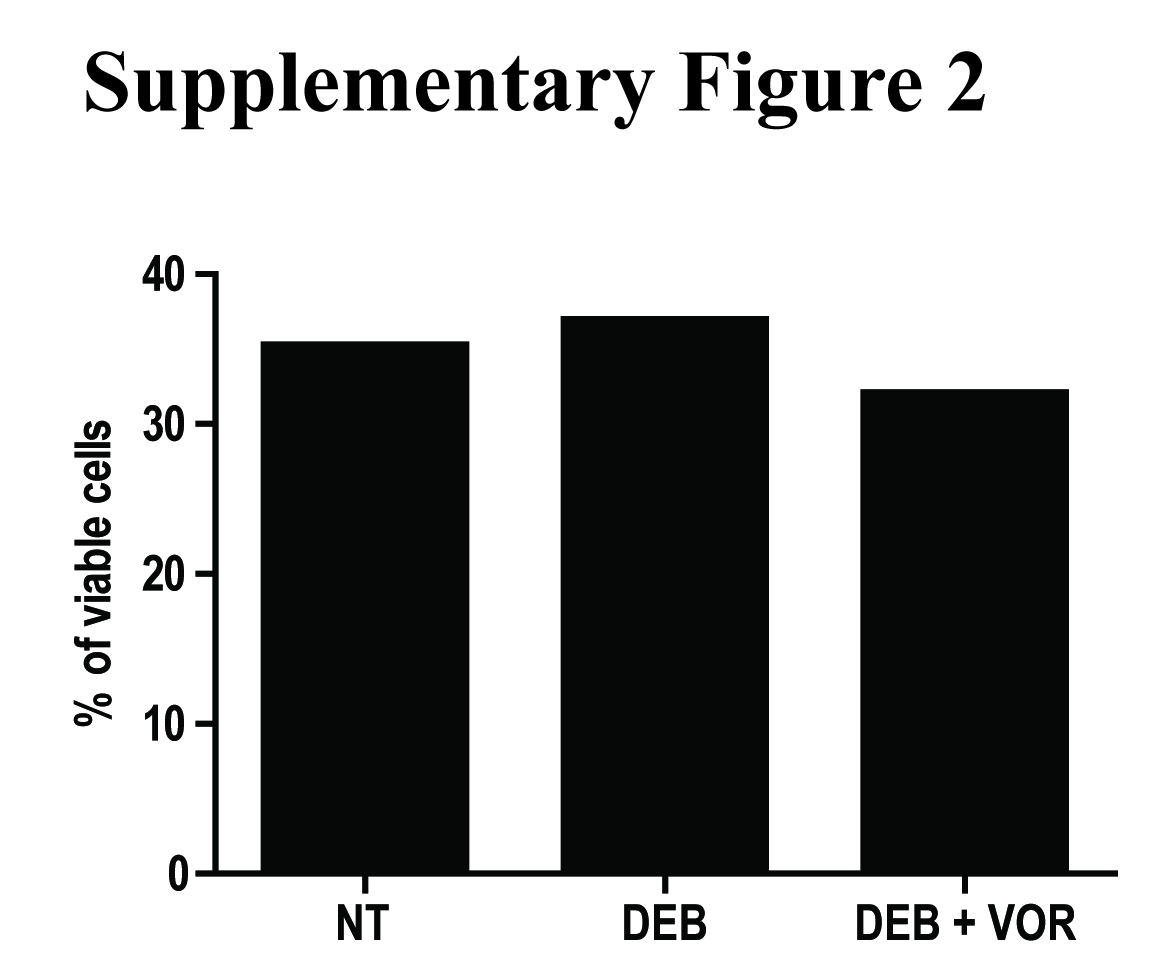

Supplement: S2 Fig — Viability PBMC of FA patients as determined by Annexin V/PI staining following treatment with Vorinostat and DEB in cell culture medium as used to test for chromosomal fragility. (TIF) [file pone.0139740.s002.tif]
